# Low-Energy Electron Irradiation Inactivates Viruses in Serum and Maintains Growth-Promoting Properties in Cell Culture

**DOI:** 10.1089/apb.2024.0031

**Published:** 2025-08-29

**Authors:** Jasmin Fertey, Gustavo R. Makert, Philipp Becker, Julia Finkensieper, Sebastian Ulbert

**Affiliations:** Fraunhofer Institute for Cell Therapy and Immunology, Leipzig, Germany.

**Keywords:** electron beam, low-energy electron irradiation, sterilization, serum, blood derived products, viral clearance

## Abstract

**Background:**

Serum and other blood-derived products are widely used in biomedical and biopharmaceutical processes, especially for the production of vaccines or cell therapeutic applications. To ensure quality and safety, each serum lot undergoes testing for sterility to minimize the risk of disease transmission. A currently performed standard procedure is gamma-irradiation of serum for effectively killing pathogens. However, gamma irradiation can only be performed in highly shielded, specialized facilities. Low-energy electron irradiation (LEEI) is an alternative, Good Manufacturing Practice (GMP) compatible method for sterilizing serum in a standard laboratory environment.

**Results:**

We directly compared the sensitivity of viruses belonging to the family of flaviviruses (Zika virus and bovine viral diarrhea virus) and parvoviruses (minute virus of mice) against LEEI in 90% serum or buffer. Although the reduction is more effective in buffer than in serum, the calculated decimal reduction dose values suggest that LEEI can efficiently sterilize serum by inactivating viruses, thereby increasing product safety and extending shelf life. Furthermore, LEEI-treated serum was tested as a supplement in cell culture and showed comparable performance to nonirradiated or gamma-irradiated serum when used for the cultivation of different cell lines.

**Conclusion:**

LEEI is able to inactivate viral contaminants in serum and offers significant advantages over gamma irradiation, as no large amounts of radioactivity are generated, on-site irradiation is possible, and space requirements are reduced due to fewer protective measures. These advantages allow the technology to be used in a GMP-compliant irradiation process.

## Introduction

Serum and other blood-derived products are necessary components in biomedical and biopharmaceutical production processes. They are commonly used in biomedical research, diagnostic kits, cell culture-based test systems for pharmaceuticals and cosmetics, as well as in growth media for the production of therapeutics (such as antibodies) and vaccines. However, the potential risk of disease transmission through biological materials exists and requires mitigation strategies. In the past, viral contamination of biologics has resulted in the transmission of infectious viruses to patients (e.g., transmission of hepatitis C virus and human immunodeficiency virus type 1) through human-plasma-derived biological products.^1,2^

The major risks for viral contamination in cell culture for therapeutic production are cell sources, materials used in cell culture, and exposure of the cell culture process stream to the operator or environment.^3^ Consequently, viral clearance studies are an essential prerequisite for ensuring safety in biopharmaceutical manufacturing processes.^3,4^ Regulatory bodies mandate virus testing to confirm the absence of viral agents.^5–8^ To mitigate contamination risks, manufacturers select low-risk materials (e.g., human serum from selected, pretested donors), conduct in-process testing to reject contaminated batches, or implement virus clearance methods such as filtration, UV irradiation, and heat treatment. These clearance methods aim to reduce virus titers significantly, typically by 3–4 log units.^4,9–11^

Ionizing radiation, such as gamma or X-ray irradiation, is currently the most commonly employed final sterilization method for pathogen reduction in animal serum. Usually, 0.5- to 1-L bottles of serum are irradiated in a frozen state. This procedure is performed in specialized irradiation facilities, due to the requirement of large radiation shielding constructions. Ionizing radiation, including gamma rays, X-rays, or electrons, has been utilized for decades to deactivate pathogens. This sterilization practice has become common not only for serum but also for food or packaging. The materials are exposed to electrons or photons, resulting in the disruption of atomic bonds and the generation of radicals. The mechanism of virus inactivation by ionizing irradiation is based on direct and indirect effects. Direct effects are mainly caused by radiolytic cleavage or crosslinking of the nucleic acid. Indirect effects rely on the actions of free radicals such as ·OH, which are created from the radiolytic cleavage of water, and to the actions of ozone, created from the radiolytic cleavage of O_2_ to O and its subsequent reaction with another O_2_ molecule. These molecules can react with viral nucleic acids as well as proteins. The destruction of replication-competent nucleic acids, via both direct and indirect mechanisms, is believed to be the major mechanism of virus inactivation by ionizing radiation.^12^ The indirect effects of irradiation are dampened by radical scavengers, which are molecules such as proteins that react with hydroxyl radicals and ozone, thereby inhibiting their ability to act on viral nucleic acids or proteins. These scavengers are of particular importance for the irradiation of serum, which is highly enriched in soluble proteins.

We tested low-energy electron irradiation (LEEI) for the inactivation of different viruses in serum. The LEEI technique is a nonthermal inactivation method for pathogens, which can be applied to the irradiation of liquids in an automated process, compatible with GMP requirements of biopharmaceutical manufacturing facilities. LEEI has several advantages because of its high dose rate, which means that the time to complete application of the dose is very short and it generates only limited amounts of secondary photon radiation (Bremsstrahlung, X-rays). Therefore, it requires less radiation shielding, which enables its use in normal laboratories. We have demonstrated the applicability for several pathogens and were able to inactivate highly infectious, pathogen-containing solutions by LEEI in order to use them as vaccine antigens.^13–16^ However, the pathogens for these experiments were in defined buffered solutions and not in complex, high-protein-containing matrices.

To investigate whether LEEI is also suitable for the depletion of viral contaminants in serum, we chose three different model viruses: Zika virus (ZIKV), bovine viral diarrhea virus (BVDV), and parvoviruses (minute virus of mice [MVM]) for testing their sensitivity toward LEEI in serum and phosphate-buffered saline (PBS). They belong to virus families that are often associated with contamination of biological material or represent pathogen families that are involved in regular testing. BVDV is a single-stranded RNA virus that belongs to the pestivirus genus and is a widely known contaminant of bovine serum.^17^ Similar to hepatitis C virus, ZIKV belongs to the *Flavivirus* genus and is a possible contaminant in human serum. MVM belongs to the *Parvoviridae* family and is a common infection in laboratory mice due to its highly contagious nature.^18^ Parvoviruses are small, nonenveloped viruses that are very stable in the environment and resistant to inactivation from heat, desiccation, and many common disinfectants.

## Results

**Figure 1.**
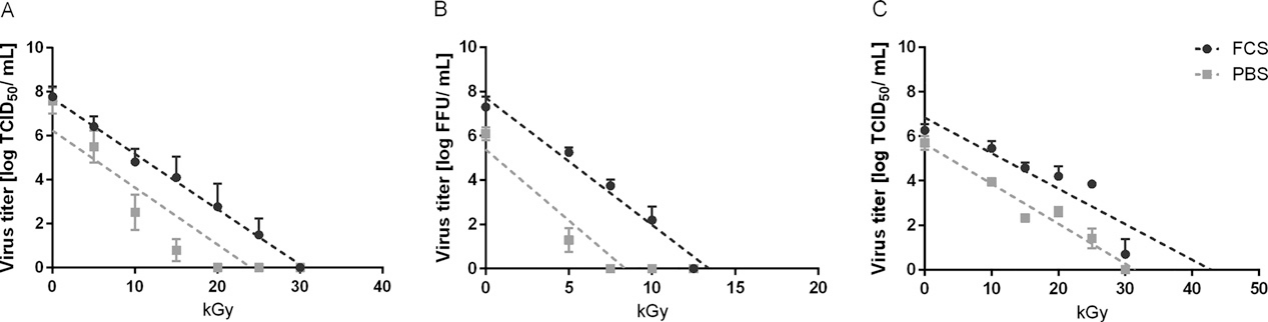
Dose curves for **(A)** ZIKV, **(B)** BVDV, and **(C)** MVM. Virus titers were determined (TCID50/mL or FFU/mL) for each matrix (FCS: black dots, PBS: grey squares) and mean values ± SEM of different irradiation experiments (*n* = 3) were plotted against the applied dose in kilogray (kGy). Fitting lines were done by linear regression and are depicted as dashed line. BVDV, bovine viral diarrhea virus; FCS, fetal calf serum; MVM, minute virus of mice; PBS, phosphate-buffered saline; ZIKV, Zika virus.

The inactivation of viruses by LEEI has been described to follow linear dose curves,^19^ which may, however, be influenced by the composition of the medium. In order to directly compare the inactivation kinetics in serum and buffer, we spiked high-titer virus solutions in either 90% PBS or 90% fetal calf serum (FCS) (final concentration) as the matrix. For each virus, three independent irradiation experiments (each in duplicates) were performed at ambient temperature. Subsequently, the residual virus titer was determined for each applied dose by 50% tissue culture infectious dose (TCID50) or focus-forming units (FFU) assay and plotted against the respective dose in kilogray (kGy). A dose-dependent reduction in titer was demonstrated for each of the tested viruses, although differences between the matrices were observed (Figure 1). For all three viruses, a higher dose was required to reduce the viral titer in 90% FCS compared with 90% PBS. For ZIKV, a dose of 20 kGy resulted in no measurable titer after irradiation in PBS, whereas in FCS at least 30 kGy was necessary (Figure 1A). For BVDV in PBS, 5 kGy resulted in a virus titer below the detection limit (100 infectious virus particles/mL), compared with 10 kGy in the FCS-spiked sample (Figure 1B). As already described in earlier studies with parvoviruses, 25 and 30 kGy were necessary to reduce MVM titers below the detection limit of 1E + 02/mL in PBS and FCS, respectively (Figure 1C). Linear regression analysis was performed and the negative reciprocal of the slope was calculated and used to determine the decimal reduction dose (D10) value for each virus. The D10 value represents the dose that is required to achieve a 90% (1-log) titer reduction of the respective target virus.

This calculation resulted in different D10 values for FCS and PBS, which corresponds to the respective slopes in Figure 1. The differences between the inactivation kinetics in FCS and PBS were confirmed for all three viruses by regression slope analysis by *p* < 0.0001 (Table 1).

**Table 1.** Mean Starting Titers (log10 per mL), Linear Regression Results, Statistics, and D10 Values in Kilogray for the Different Viruses in 90% Fetal Calf Serum or Phosphate-Buffered Saline as Matrix

Virus	ZIKV		BVDV		MVM	
Matrix	FCS	PBS	FCS	PBS	FCS	PBS
Starting titer (mean ± SD)	7.78 ± 1.15	7.24 ± 1.95	7.30 ± 0.92	6.09 ± 0.56	6.12 ± 0.66	5.87 ± 0.94
Best-fit values						
Slope	−0.2519 ± 0.02452	−0.2593 ± 0.02557	−0.5747 ± 0.04133	−0.6417 ± 0.08107	−0.1597 ± 0.02069	−0.1795 ± 0.01156
Y-intercept X = 0.0	7.689 ± 0.4420	6.234 ± 0.4609	7.722 ± 0.3396	5.383 ± 0.5061	6.839 ± 0.4006	5.659 ± 0.2239
X-intercept Y = 0.0	30.53	24.04	13.44	8.389	42.83	31.52
1/slope	−3.971	−3.856	−1.740	−1.558	−6.262	−5.570
95% Confidence intervals						
Slope	−0.3014 to −0.2023	−0.3110 to −0.2076	−0.6602 to −0.4892	−0.8144 to −0.4689	−0.2018 to −0.1176	−0.2031 to −0.1560
Y-intercept X = 0.0	6.796–8.582	5.302–7.165	7.020–8.425	4.305–6.462	6.024–7.654	5.204–6.115
X-intercept Y = 0.0	27.40–34.91	21.65–27.18	12.41–14.76	7.219–10.09	36.90–52.66	29.41–34.15
Goodness of fit						
R square	0.7251	0.7200	0.8937	0.8068	0.6367	0.8764
Line differences FCS vs. PBS						
*F*	19.8035		65.245		19.835	
DFn. DFd	1.000. 81.00		1.000. 39.00		1.000. 81.00	
*p* value	<0.0001		<0.0001		<0.0001	
Equation	*Y* = −0.2519*X + 7.689	*Y* = −0.2593*X + 6.234	*Y* = −0.5747*X + 7.722	*Y* = −0.6417*X + 5.383	*Y* = −0.1597*X + 6.839	*Y* = −0.1795*X + 5.659
D10 value	3.97 kGy	3.86 kGy	1.74 kGy	1.56 kGy	6.26 kGy	5.57 kGy

BVDV, bovine viral diarrhea virus; FCS, fetal calf serum; MVM, minute virus of mice; PBS, phosphate-buffered saline; ZIKV, Zika virus.

**Figure 2.**
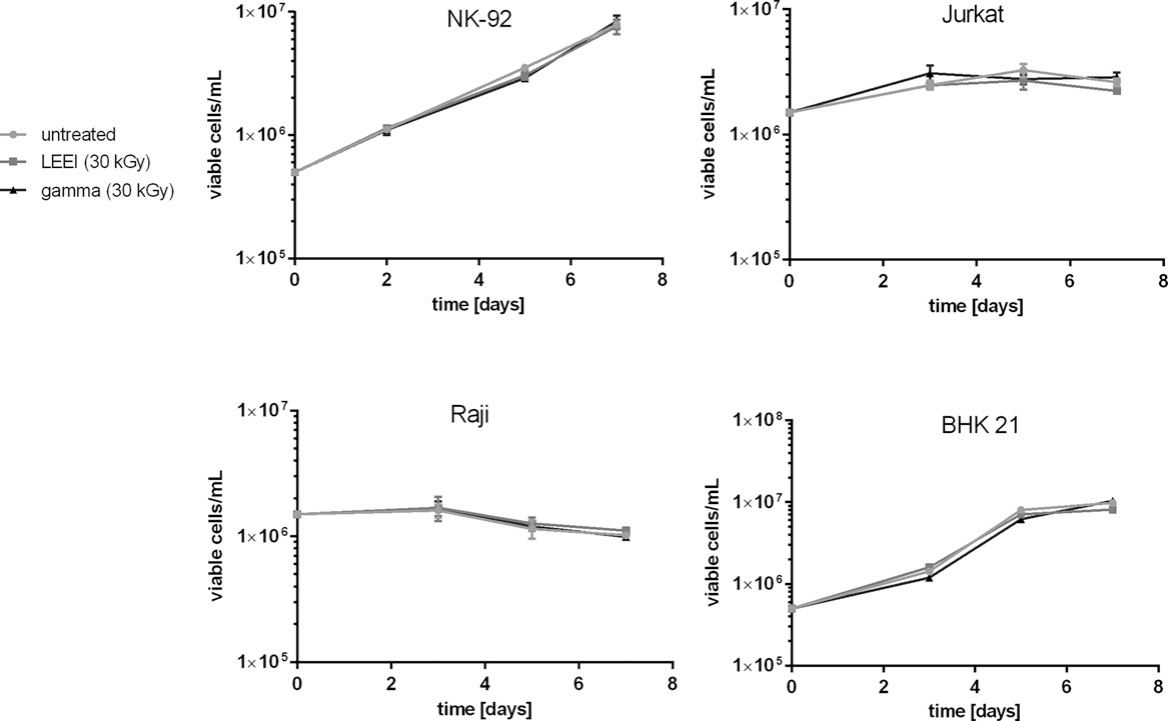
Growth curves for different cell lines with LEEI (grey squares) or gamma (black triangles) treated serum. Serum was irradiated with a dose of 30 kGy and added to the medium in a final concentration of 10%. Untreated serum served as positive control. Cell counting was performed in duplicates, graphs show mean values of the counting with standard deviations. LEEI, low-energy electron irradiation.

To test the performance of irradiated serum for the cultivation of mammalian cells, FCS was irradiated with either 30 kGy LEEI or with 30 kGy gamma radiation and added to the culture medium of different cell lines in a final concentration of 10%. Untreated FCS served as a positive control. Cell growth was monitored every 2–3 days for 7 days in total by cell counting and determination of viable and dead cells. For all tested cell lines, no major differences in cell growth between untreated, LEEI- and gamma- radiation-treated serum were observed (Figure 2). Cell viability was only slightly affected (Table 2) and this effect varied between cell lines, which indicates no particularly negative effects of either LEEI or gamma treatment on the performance of serum.

**Table 2.** Viability (% ± SD) of Different Cell Lines After 7 Days When Cultured with Untreated Serum, with 30 kGy Gamma-Irradiated Serum, or with Low-Energy Electron-Irradiated Serum

Treatment	Cell line			
	NK-92	Raji	Jurkat	BHK 21
Untreated	90.51 ± 0.19	79.49 ± 5.58	81.48 ± 2.47	93.33 ± 1.15
Gamma 30 kGy	92.81 ± 0.01	69.94 ± 6.44	77.65 ± 1.09	88.83 ± 2.64
LEEI 30 kGy	86.39 ± 2.83	59.77 ± 8.18	75.77 ± 8.64	93.68 ± 1.31

LEEI, low-energy electron irradiation.

## Material and Methods

### Cell Culture

Jurkat, Raji, NK-92, BHK21, and VeroE6 cells were obtained from DSMZ Braunschweig. A9 cell line was kindly provided by Antje Rückner, Institute for Virology, Veterinary Faculty, Leipzig University. The KOP-R cell line was obtained from Friedrich Loeffler Institute. The BHK21 and VeroE6 cells were cultivated in Dulbecco’s modified Eagle medium (DMEM) (Gibco) with 10% FCS and 1% penicillin/streptomycin. The KOP-R-cells were cultivated in minimal essential medium (MEM) + 4 mM Glutamax (Gibco) with 10% FCS and 1% penicillin/streptomycin. The Jurkat and Raji cells were cultivated in Roswell Park Memorial Institute 1640 medium with 10% FCS and 1% penicillin/streptomycin. The NK-92 were cultured in MEM-alpha medium with 10% FCS (Sigma-Aldrich, USA), 1% penicillin/streptomycin (Thermo Fisher Scientific, USA), and interleukin 2 (IL-2; Proleukin S, Novartis, Germany) in a final concentration of 100 IU/mL. The A9 cells were propagated in high-glucose DMEM (ThermoFisher Scientific, Germany) + 1 mM Natrium-Pyruvat (Gibco) and 1% nonessential amino acids (Gibco), supplemented with 10% heat-inactivated FCS (Gibco) and penicillin (100 U/mL) and streptomycin (100 μg/mL). All cell lines were maintained at 37°C and 5% CO_2_.

For growth curves, cells were seeded in six-well plates and the total number of cells was counted using a Neubauer counting chamber every 2–3 days. Viability was assessed by staining with trypan blue.

### Virus Culture and Titration

ZIKV was cultivated on VeroE6 cells and purified over a sucrose cushion by ultracentrifugation as previously described.^20^ In brief, ZIKV (Padova; Dominican Republic/2016/PD1; GenBank KU853012, kindly provided by Luisa Barzon, Padova University, Padova, Italy) was purified by ultracentrifugation through a sucrose cushion and viral pellets were resuspended in PBS containing 10% (w/v) sucrose (Carl Roth GmbH, Germany). The purified virus was stored in aliquots at −80°C until use. ZIKV titers were determined with end-point dilutions via the TCID50 assay, by adding 10-fold dilutions of viral stocks to Vero E6 cell monolayers and incubation for 5 days at 37°C and 5% CO_2_. The cells were monitored for cytopathic effect (CPE) and the titer was calculated using the Reed–Muench method.^21^

The BVDV strain 1a NADL (Friedrich-Loeffler-Institute, RVB-0201) was cultivated on KOP-R cells. The KOP-R cells were cultured to a 70–80% confluency and were infected with BVDV at a multiplicity of infection (MOI) of 0.5. After 2–3 days at 37°C and 5% CO_2_, a CPE could be observed and the virus-containing cell culture supernatant was harvested. After removing cell debris, the virus particles were concentrated by ultracentrifugation using a 20% sucrose cushion for 3 h at 4°C and 80,817 rcf using an Ultracentrifuge Rotor Sorvall SureSpin 630 (Thermo Fischer Scientific, USA). Afterward, the supernatant was discarded and the pellet was resuspended in 20% sucrose PBS solution.^22^ The purified BVDV was cryo-conserved in liquid nitrogen or stored at −80°C until the virus titration. The BVDV titers were determined by FFU assay. In brief, 40,000 KOP-R cells were seeded into a 96-well plate (Greiner Bio-One GmbH, Germany) and infected with 10-fold dilutions of BVDV stock on the following day. After 1 h of incubation at 37°C, the KOP-R cells were covered with an overlay medium (1% methylcellulose in DMEM supplemented with 2% FCS) and incubated for 48 h at 37°C and 5% CO_2_. After fixation with 4% formaldehyde in 1× PBS (Carl Roth GmbH, Germany) for 20 min, the KOP-R cells were permeabilized and blocked with PBS containing 1 g saponin (Carl Roth GmbH, Germany) and 1 g bovine serum albumin (Carl Roth GmbH, Germany), before incubation steps with mouse antipestviruses antibody (APHA Scientific, UK, diluted 1:5000) for 2 h at room temperature (RT), and then with rabbit horseradish peroxidase antimouse antibody (Agilent, USA, diluted 1:1500) for 1 h at RT. After washing, the spots were detected after 20 min of incubation with TrueBlue substrate (KPL, Seracare, USA) in the CTL ImmunoSpot Series 6 universal Analyzer (Cellular Technology Limited, CTL Europe).

MVM (kindly provided by Antje Rückner, Institute for Virology, Veterinary Faculty, Leipzig University) was cultivated in A9 cells. Cells were cultured to a confluence of 50–60% and infected with an MOI of 0.1. After 3–4 days of incubation (37°C and 5% CO_2_) or until the CPE was visible in the culture, the remaining cells were scraped of the culture flask and collected with the virus-containing supernatant. Cells were disrupted by three freeze-and-thaw cycles to release additional virus into the supernatant. The MVM was purified from the supernatant by ultracentrifugation (78,109 rcf) on a CsCl-cushion (1.4 mg/mL in 0.2M Tris pH 7.5) for 2.5 h at 4°C. The MVM was titrated using the TCID50 assay on A9 cells. In short, serial 10-fold dilutions of viral stocks were added to A9 cell monolayers with a 50% confluence in a 96-well microwell plate and incubated for 6 days. The cells were monitored for CPE and the titer was calculated using the Reed–Muench method.^21^

### Irradiation

Virus samples were irradiated as previously described.^13^ Virus stocks were inoculated into PBS or FCS to achieve an inoculation level between 10% virus and 90% matrix. In short, concentrated virus stock was diluted 1:10 in either PBS or FCS. Two hundred and thirty microliters of the virus suspension were applied to the center of a sterile 100-mm Petri dish (Primaria^TM^, Corning, NY, USA). To generate a liquid film of approximately 100 µm thickness, the suspension was overlaid with a round oriented polypropylene-foil (50 mm diameter). Irradiation was performed in duplicates with 10, 15, 20, 25, or 30 kGy with a 200 keV electron beam at RT (Comet, EB-Lab200, Switzerland). Afterward, the liquid was recovered from the Petri dish by carefully tapping and removing the liquid with a pipette. Approximately 65% (150 µL) of each sample was recovered and used for further analyses.

The serum for growth curves was sealed in plastic bags and irradiated with 30 kGy LEEI using a customized research prototype as described by Fertey et al.^15^ Gamma irradiation of serum with 30 kGy was performed at BBF Sterilisationsservice GmbH, Kernen im Remstal, Germany, using a Cobalt-60-radiation source in a frozen state on dry ice.

### Data Analysis

Data were plotted and analyzed using GraphPad Prism (Version 6.0.7 [GraphPad Software]) with the log (10) values of the virus titers on the *y*-axis against the corresponding radiation doses on the *x*-axis. Linear regression was used to fit a line through the data points. D10 values were quantified by computing the inverse slope of the regression line best-fitting the dose curve of each virus kGy versus TCID50 or FFU. The fitting lines of the corresponding PBS and FCS samples were analyzed for differences by comparing the Y-intercept and slope. Best-fit values, standard errors, and 95% confidence intervals were calculated from individual replicates along with the goodness of fit with *p* < 0.05.

## Discussion

Ionizing radiation can sterilize serum or other biological liquids by inactivating bacteria, viruses, and fungi, thus improving product safety and extending shelf life.^10,23–25^ Especially in the emerging field of production of advanced therapy medicinal products, virus contamination can have severe effects for patients, either by the contamination itself or by delays in administering treatment. A possible option to reduce the contamination risk is the treatment of high-risk raw materials, such as, sera or growth supplements, with irradiation.^24,26^ While it is not yet a standard practice for human serum, it is applied for animal serum products. Currently used procedures for sterilization involve mainly gamma irradiation with doses between 25 and 50 kGy to deplete adventitious reagents, such as viruses or mycoplasma.^25^ It has been described that the efficacy of virus deactivation through irradiation largely depends on the matrix and temperature with the mechanism of inactivation varying based on the existing conditions during the process.^10^ While some studies report that proteins in solution have a negative impact on gamma inactivation,^27–29^ other studies suggest that protein content does not alter the efficacy of gamma inactivation, particularly on frozen samples.^27,30^

A major challenge in mitigating the risk of adventitious agents through irradiation is the potential negative impact on the serum quality and performance for downstream applications, such as cell culture. With a standard dose of 30 kGy, which is also used for gamma treatment of commercially available FCS, we could not observe any major differences between gamma-, LEEI-, or nonirradiated serum in the growth characteristics of four different cell lines (BHK 21, Jurkat, Raji, and NK-92 cells). This observation is in line with other studies, in which gamma irradiation of serum with 45 kGy had no negative effects on the growth of WI-38 cells and MRC-5 cells, although other Vero cells or CHO cells showed reduced growth at 15–45 kGy.^31^ Others reported that cell growth was even supported for 3T3 cells, MDBK cells, and L243 cells with gamma-irradiated serum at 25–35 kGy compared with nonirradiated serum.^23,32^

Our results are in line with previous studies, indicating that irradiation of serum is effective for inactivation of larger enveloped and nonenveloped viruses.^25,33,34^ The effectiveness is evaluated via the number of log units (of the virus titer) that is reduced through the procedure. For these assays, artificially high virus titers (10^6^/mL or higher) are used, which do not correspond to the real amount present in a viral contamination event. These high titers are employed to be able to reproducibly measure virus depletion. While BVDV in serum is relatively sensitive toward electron irradiation, with described D10-values of 2.5 kGy^35^ and 1.74 kGy (this study), we determined a D10 of 3.97 kGy for ZIKV. The effectiveness against smaller, nonenveloped viruses like the porcine Parvovirus and MVM is significantly less,^25,35,36^ which was also confirmed in this study. For MVM, we determined a D10 of 6.20 kGy in serum, which is lower compared with published D10 values for gamma irradiation of 10.7 kGy or approximately 15 kGy.^32,37^ Nevertheless, in contrast to the mentioned studies involving gamma irradiation, LEEI at 30 kGy was sufficient to achieve a titer reduction of at least three log units of MVM, representing a suitable efficacy. In general, the D10 values obtained here and in similar studies indicate that for many viruses, a dose less than 30 kGy (the standard sterilizing dose for gamma irradiation required by authorities) would be sufficient for inactivation. However, since some virus families are more resistant, a high dose of 30kGy is warranted to assure biosafety in the absence of information on the viruses that are actually present in the sample. A difficulty in comparing D10 values obtained in different studies is that the irradiation procedures are not standardized and that other influences such as temperature also play an important role. Overall D10 values usually differ in a direct comparison between frozen and liquid material. Preuss et al.^35^ reported a D10 for MVM of 11.8 kGy in frozen bovine serum and 7.7 kGy in liquid serum using e-beam radiation. Another study using gamma irradiation and cell culture supernatant described D10 values of 4.2 kGy (frozen, dry ice), 1.2 (liquid, ice), and 0.9 kGy (liquid, RT).^38^ These results suggest that irradiation of liquids generally requires a lower dose to reduce pathogens than of frozen material.

Since LEEI is a process optimized for liquids, it provides a suitable alternative to gamma irradiation for virus reduction in serum and other biological products. The results indicate that LEEI can be used to efficiently reduce virus titers at least 3–4 log levels in FBS and that the treatment does not interfere with the ability of the serum to promote cultivation of different cell lines. The LEEI technique has significant advantages over gamma irradiation. It does not generate large amounts of radioactivity. Therefore, irradiation could be performed on site, either at the facility obtaining the material or in the pharmaceutical production facility ensuring complete traceability, even when applying high doses as required in viral clearance. This makes it possible to use them directly in biological production laboratories, as the space requirement is significantly lower due to reduced protective measures. For the perspective of industry-scale production, automated processes have been developed that enable continuous application of LEEI for multiliter throughputs.^15^ The application of the target dose is very fast in LEEI, minimizing the exposure time of the liquid and thereby the induction of indirect irradiation effects, that is, beneficial for product integrity. This feature would allow for a GMP-compliant process and thus offer a clear advantage over other irradiation methods.
